# Two Trackers Are Better than One: Information about the Co-actor's Actions and Performance Scores Contribute to the Collective Benefit in a Joint Visuospatial Task

**DOI:** 10.3389/fpsyg.2017.00669

**Published:** 2017-05-03

**Authors:** Basil Wahn, Alan Kingstone, Peter König

**Affiliations:** ^1^Institute of Cognitive Science, Universität OsnabrückOsnabrück, Germany; ^2^Department of Psychology, University of British ColumbiaVancouver, BC, Canada; ^3^Institut für Neurophysiologie und Pathophysiologie, Universitätsklinikum Hamburg-EppendorfHamburg, Germany

**Keywords:** Social cognition, visuospatial attention, collective benefit, joint action, Multiple object tracking

## Abstract

When humans collaborate, they often distribute task demands in order to reach a higher performance compared to performing the same task alone (i.e., a collective benefit). Here, we tested to what extent receiving information about the actions of a co-actor, performance scores, or receiving both types of information impacts the collective benefit in a collaborative multiple object tracking task. In a between-subject design, pairs of individuals jointly tracked a subset of target objects among several moving distractor objects on a computer screen for a 100 trials. At the end of a trial, pairs received performance scores (Experiment 1), information about their partner's target selections (Experiment 2), or both types of information (Experiment 3). In all experiments, the performance of the pair exceeded the individual performances and the simulated performance of two independent individuals combined. Initially, when receiving both types of information (Experiment 3), pairs achieved the highest performance and divided task demands most efficiently compared to the other two experiments. Over time, performances and the ability to divide task demands for pairs receiving a single type of information converged with those receiving both, suggesting that pairs' coordination strategies become equally effective over time across experiments. However, pairs' performances never reached a theoretical limit of performance in all experiments. For distributing task demands, members of a pair predominantly used a left-right division of labor strategy (i.e., the leftmost targets were tracked by one co-actor while the rightmost targets were tracked by the other co-actor). Overall, findings of the present study suggest that receiving information about actions of a co-actor, performance scores, or receiving both enables pairs to devise effective division of labor strategies in a collaborative visuospatial task. However, when pairs had both types of information available, the formation of division of labor strategies was facilitated, indicating that pairs benefited the most from having both types of information available (i.e., actions about the co-actor and performance scores). Findings are applicable to circumstances in which humans need to perform collaborative visuospatial tasks that are time-critical and/or only allow a very limited exchange of information between co-actors.

## 1. Introduction

In everyday life, humans often perform tasks collaboratively that otherwise would be too difficult or cumbersome to perform alone. In such joint tasks, humans coordinate their actions in space and time in order to achieve a shared goal (i.e., a change in the environment; for general reviews, see: Sebanz et al., 2006; Frith and Frith, 2012; Vesper et al., 2016a). For instance, when two people are searching for a friend in a large crowd, one person may focus his search on the left half of a crowd while the other person searches the right half of the crowd (Brennan et al., 2008). Such a distribution of task demands between co-actors enables groups to reach a higher performance than their individual performances (i.e., a collective benefit) (Brennan et al., 2008; Bahrami et al., 2010).

Collective benefits have been researched extensively in the past in several domains such as decision-making (Bahrami et al., 2010, 2012a,b), attention (Brennan et al., 2008; Neider et al., 2010; Wahn et al., 2016c; Brennan and Enns, 2015), or sensorimotor processing (Knoblich and Jordan, 2003; Masumoto and Inui, 2013; Ganesh et al., 2014; Rigoli et al., 2015; Skewes et al., 2015; Wahn et al., 2016b). This work has converged on the conclusion that several factors may influence if, and to what extent, groups outperform individuals (Knoblich and Jordan, 2003; Brennan et al., 2008; Bahrami et al., 2010).

One of these factors is the type of information that is exchanged between co-actors (Brennan et al., 2008; Neider et al., 2010; Wahn et al., 2016c). For instance, in a study by Brennan et al. (2008), the type of exchanged information systematically affected collective benefits in a collaborative visual search task. In particular, in their study, participants performed a search task either alone or in pairs. While they searched together, they were either not permitted to communicate or they were allowed to communicate in one of three ways: verbally, by seeing a cursor on the screen indicating where their search partner was looking, or both, verbally and by seeing the cursor. While Brennan et al. (2008) generally found that pairs outperformed individuals, the most efficient search performance was achieved when pairs only received their partner's gaze information (i.e., information where their search partner was looking). In this condition, pairs effectively divided the search space into two parts that only minimally overlapped, enabling them to require only half of the time individuals needed to complete the search. These findings generally suggest that the collective benefit in visuospatial tasks such as collaborative visual search depends on an effective exchange of information about the performed actions of co-actors. In this particular case, it is an effective exchange of gaze information that enables co-actors to efficiently perform the collaborative visual search task. However, there are questions related to collaborative visuospatial tasks that have not been investigated, yet. Specifically, it has not been investigated to what extent receiving information about the performance accuracy (e.g., whether trials were correctly classified as target present or not present in a joint visual search task) contributes to the collective benefit, as this aspect of the task was not manipulated experimentally in earlier studies (Brennan et al., 2008; Neider et al., 2010; Wahn et al., 2016c). Additionally, it has not been investigated to what extent exchanging information about the co-actors' actions by itself contributes to the collective benefit.

While the contribution of the performance accuracy to the collective benefit has not been researched in collaborative visuospatial tasks, its contribution has been investigated in the domain of collaborative decision-making (Bahrami et al., 2010, 2012a). In particular, researchers investigated to what extent receiving performance scores, verbal communication, or both (i.e., performance scores as well as verbal communication) can predict a collective benefit in a collaborative visual discrimination task (Bahrami et al., 2010, 2012a). Results showed that participants reached the highest collective benefit when they were allowed to communicate and received performance scores. They still reached a collective benefit when they were only allowed to communicate with each other but when only performance scores were provided, no collective benefit was achieved. Notably, an analysis of the verbal communication showed that pairs who were linguistically aligned (i.e., used similar linguistic practices) showed a greater collective benefit (Fusaroli et al., 2012; Fusaroli and Tylén, 2016). In sum, pairs in a collaborative decision-making task can reach a collective benefit when they verbally negotiate their joint decisions. Importantly, this collective benefit is further increased when also having performance scores available, suggesting that performance scores in combination with other information can facilitate reaching a collective benefit.

Taken together, previous studies investigating collective benefits in collaborative visuospatial tasks showed that exchanging information about the co-actors' performed actions leads to a high collective benefit (Brennan et al., 2008; Neider et al., 2010; Brennan and Enns, 2015; Wahn et al., 2016c). Other studies investigating collective benefits in a collaborative decision-making task showed that having performance scores available about the individual and co-actors' decisions can further increase an already existing collective benefit (Bahrami et al., 2010, 2012a). To date, however, researchers have not investigated to what extent receiving information about the co-actor's performed actions, receiving performance scores, or both contributes to the collective benefit in a collaborative visuospatial task.

In the present study, three experiments tested how information on the performed actions of a co-actor, performance feedback, or both, contribute to the collective benefit in a multiple object tracking (“MOT”) task (Pylyshyn and Storm, 1988) that is performed together. As a point of note, human performance in a MOT task has predominantly been studied in isolation (Cavanagh and Alvarez, 2005; Alvarez and Franconeri, 2007; Wahn and König, 2015a,b; Wahn et al., 2016a, 2017). To the best of our knowledge, the present study is the first to investigate collaborative behavior of two individuals in a jointly performed MOT task. In a MOT task that is performed alone, participants first see several stationary objects on a computer screen and a subset of these objects are indicated as “targets.” Then, objects become indistinguishable and move across the screen in random directions for several seconds and participants are instructed to track the movements of the targets. When objects stop moving, participants are required to select which objects were the targets and then typically receive information about their performance (i.e., whether objects were correctly selected or not). We chose the MOT task for the present study as it allows a quantification of performance scores (i.e., correctly selected objects). In addition, the exchange of information about the actions of co-actors (i.e., the selected objects) can be precisely controlled. Moreover, the MOT task is a highly demanding visuospatial task if it is performed by one individual (Alvarez and Franconeri, 2007; Wahn et al., 2016a), potentially motivating the need for co-actors to divide task demands. Finally, the MOT task does allow to divide task demands – for instance, one co-actor could decide to track one subset of targets while the other co-actor could decide to track the complementary set of targets.

In the collaborative version of the MOT task designed for the present study, two participants perform the MOT task at the same time. In particular, both participants receive the same target indications and see identical object movements on their individual computer displays. Once objects stop moving, members of a pair individually select the objects that they think are the targets. Then, in Experiment 1, pairs receive performance scores that are composed of the individual tracking performance scores and the pairs' total performance score (Experiment 1). That is, members of a pair receive feedback on how well they performed individually (i.e., whether their target selections were correct or not) and also how well they performed jointly as a pair (i.e., whether the pair's combined target selections were correct or not). In Experiment 2, pairs receive information about which objects were selected by their co-actor but no performance scores. In Experiment 3, both, performance scores (i.e., both individual and pair performance scores) and information about the partner's selections are available to the pairs.

We hypothesized that all types of provided information would separately and in combination lead to collective benefits. That is, a pair should reach a higher performance than either of the individuals constituting the pair. In particular, given earlier research on collective decision-making (Bahrami et al., 2010, 2012a), we hypothesized that having performance scores about the individual and pair's performance (Experiment 1) enables members of a pair to adjust their behavior on a trial-by-trial to devise an effective collaborative strategy. In line with earlier findings on collaborative visual search (Brennan et al., 2008; Neider et al., 2010; Brennan and Enns, 2015; Wahn et al., 2016c), when having information about the partner's object selections available (i.e., information about the actions of a co-actor – Experiment 2), we hypothesized that pairs would reach a collective benefit as well. That is, we expected that co-actors can effectively distribute the number of targets that co-actors were required to track. When having both kinds of information available (Experiment 3), we predict that this would lead to the largest collective benefit as pairs can effectively distribute the number of targets and can also use the performance scores to verify whether their division of labor strategies are effective, further enhancing the collective benefit (Bahrami et al., 2010, 2012a).

## 2. Methods

### 2.1. Participants

A total of 96 students (66 female) were recruited as participants at the University of Osnabrück and the University of British Columbia. Participants were evenly distributed across the three experiments. For each experiment, 32 students were grouped in 16 pairs (Experiment 1: *M* = 24.19 years, *SD* = 4.73; Experiment 2: *M* = 21.13 years, *SD* = 2.85 years; Experiment 3: *M* = 22.53 years, *SD* = 3.88 years). Experiments 1 and 3 were conducted at the University of Osnabrück while Experiment 2 was conducted at the University of British Columbia. Participants either received course credits or a monetary compensation for their participation. All participants had normal or corrected to normal vision. The study was approved by the ethics committee of the University of Osnabrück and of the University of British Columbia. Written informed consent was obtained from each participant.

### 2.2. Experimental setup

Each member of a pair was seated at a 90 cm distance in front of a computer that was concealed from the other member's computer either by a curtain or an occluder. Stimulus parameters for the multiple object tracking task (see Experimental Procedure below), screen resolution (1920 × 1000) and screen sizes (24′) were matched for the setups of all experiments. In order to minimize external noise, participants wore ear muffs throughout the whole experiment.

### 2.3. Experimental procedure

**Figure 1:**
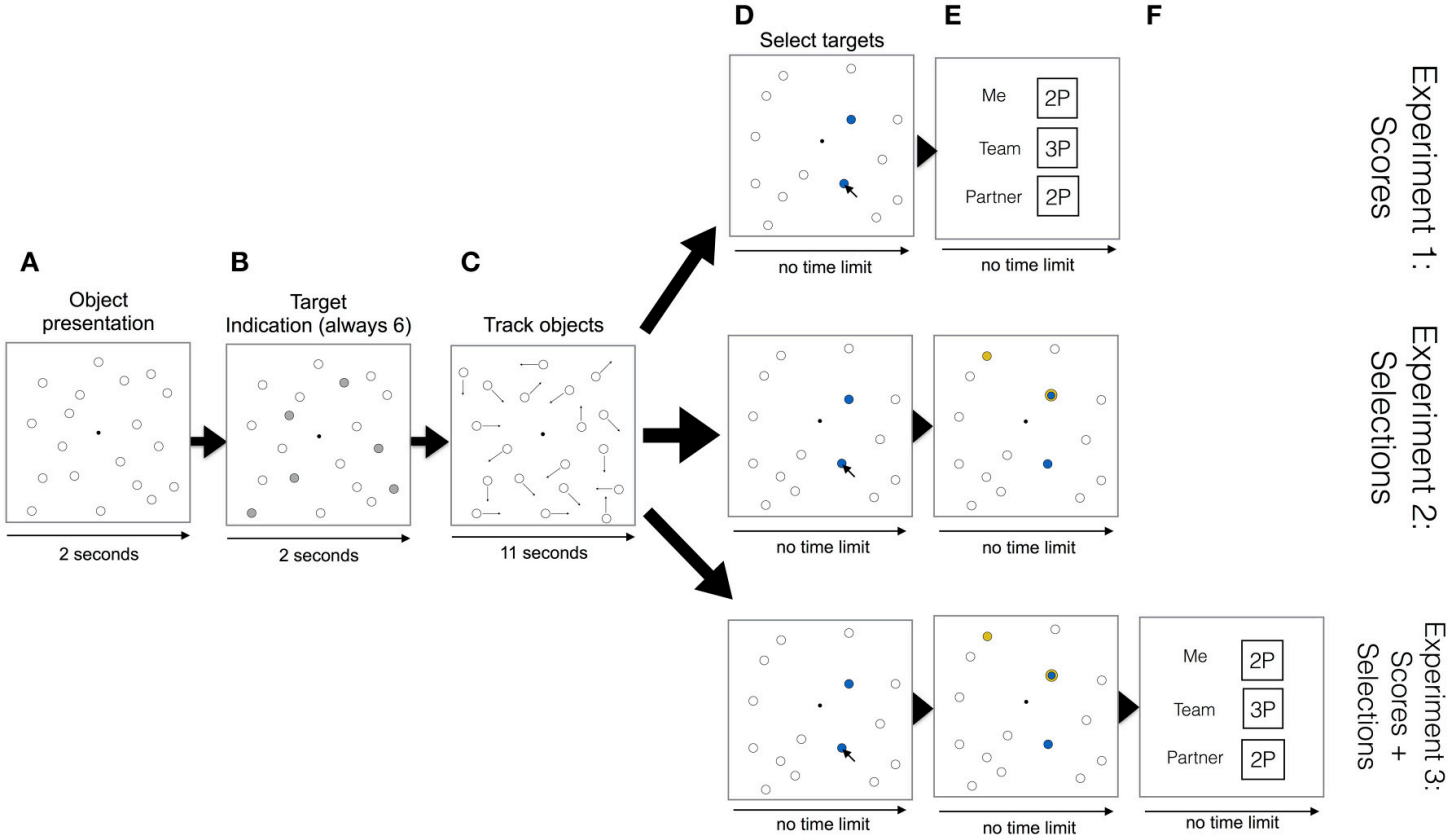
**Trial overview. (A)** Object presentation: 19 stationary white objects are presented. **(B)** Target indication: A subset of 6 targets are indicated in gray. **(C)** Track objects: Objects move across the screen bouncing off each other and from the screen borders. **(D)** Select targets: Participants individually select objects that they think are the targets. **(E)** First row: Participants receive scores about their individual performances (i.e., “Me” and “Partner”) and the pair's performance (“Team”). Second and third row: In addition to the member's own selections (shown in blue), the partner's selections are also shown (in yellow). Overlapping selections are shown in both colors. **(F)** Third row: After participants receive information on the partner's selections (and possible overlapping selections), they receive scores about their individual performances and the pair's performance.

Pairs were first verbally instructed about the experimental procedure and then one example trial was shown by the experimenter to illustrate the experimental procedure. In an experimental trial, participants first saw 19 stationary white objects (0.56 visual degree radius) for 2 s located in randomly chosen positions on the computer screen (see Figure 1A). Then, always six of these objects turned gray for 2 s (referred to as “targets,” see Figure 1B). Objects then turned white again and after an additional 0.5 s started to move in random directions across the screen for 11 s (see Figure 1C). Participants were instructed to track the movements of the targets. The object's velocity was randomly assigned to each object, varying between 0.90 and 1.21 visual degrees per second. While objects were moving, if they met the screen border or if their paths intersected they would “bounce” in a physically plausible way (i.e., angle of incidence equaled the angle of reflection). After objects stopped moving, both members selected the objects they thought were the targets. They indicated their decisions using a computer mouse (see Figure 1D).

Participants were allowed to select as many objects as they wanted. They were instructed that correctly selected target objects would add one point to their individual performance, whereas one point would be subtracted for each incorrectly selected object. Participants were also instructed that correct overlapping selections (i.e., when the same object was selected by both members of a pair) would add only one point to the pair's performance. Similarly, only one point would be subtracted from the pair's performance in the case of incorrect overlapping selections. So, for example, as depicted in 1E (first row), both members would get 2 points as their individual scores (see “Me” and “Partner”) for selecting 2 targets correctly, and as one of the correct selections overlaps, the pair's performance (see “Team”) would be 3 points.

Pairs were instructed to collaborate with the goal being to maximize the number of scored points for the pair's performance. Note, pairs were not allowed to verbally communicate throughout the whole experiment. The information exchange between members of a pair was limited to the information they received in the MOT task. Participants logged in their responses by clicking on a central black dot (0.15 visual degree radius) on the computer screen with a computer mouse. Once both members of a pair logged in their responses, depending on the experiment, different types of information were received by the participants: In Experiment 1, participants received scores about their individual performances (i.e., “Me” and “Partner”) and the pair's performance (“Team,” see Figure 1E, 1st row). In Experiment 2, pairs received information about the target selections of the partner in addition to their own selections (see Figure 1E, 2nd row). In Experiment 3, both, the partner's selections and performance scores, were received in succession (see Figure 1E,F, 3rd row). For viewing each type of information (i.e., performance scores and partner's target selections), no time limit was imposed and participants could continue whenever they felt ready for the next trial by pressing the space key on the keyboard. Whenever one of the co-actors was finished earlier than the other co-actor, pressing the space key resulted in a blank white screen being shown, which signaled to the participant to wait for their co-actor. Participants were instructed that pressing the space bar indicated that they were ready to proceed with the next trial. Once both participants had indicated that they were ready to proceed, one of the members was prompted to start the next trial by pressing the space key on the keyboard.

The experiment lasted a total of 100 trials. After the trials were completed, participants filled out a questionnaire in which they indicated whether they used a strategy to collaborate with their partner or not. If they used a strategy, participants were asked to describe the strategy in detail and whether it changed over the course of the experiment. In Experiment 3, we also asked participants whether, for developing their strategy, they relied more on the information about the target selections or the performance scores.

The experiment was programmed using Python 2.7.3, and lasted about 1 h per pair.

### 2.4. Dependent variables

For assessing whether pairs reached a collective benefit, we used several performance measures, derived measures, and theoretical limits.

First, we extracted three types of performance measures (referred to as “min,”“max,” and “pair”) for each trial. For the min and max performance, we extracted the worst and best of the two individual performances based on how many points they received on a per trial level. The pair performance is the actual performance of the pairs.

We defined the collective benefit as the difference between the pair performance and the max performance. That is, in order to test for collective benefits, we compared the difference between the max performance with the pair performance later in the analysis.

In addition, based on the correct and incorrect selections of the members of a pair, we calculated a theoretical upper and lower limit for each of the pairs' performances in a trial taking into account the individual performances. In particular, for the upper limit, we assumed that the members' correctly identified targets to be non-overlapping selections. The reasons for this choice is that, as pointed out above, a correctly selected target that both members of a pair select would add only one point to the pair's performance while two correctly selected targets that are non-overlapping would add two points to performance. Hence, treating correct target selections as non-overlapping selections maximizes the pair's performance. For incorrect target selections, we assumed that the members' selections should be overlapping selections as an incorrect overlapping selection leads to a reduction of the pair's performance by only one point compared to two points when incorrect selections would be non-overlapping. Hence, treating incorrect target selections as overlapping selections minimizes reductions of the pair's performance. In sum, this procedure (i.e., assuming non-overlapping selections for correct selections and overlapping selections for incorrect selections) maximizes the number of points for correct selections and minimizes the reductions for incorrect selections, resulting in an upper limit of performance. For the lower limit, we reversed this pattern of how correct and incorrect selections were assigned (i.e., overlapping selections for correct selections; non-overlapping selections for incorrect selections). These measures allowed us to normalize the pairs' performance within each experiment to compare performances across experiments later on.

A recent study by Brennan and Enns (2015) suggests the need for another baseline for comparison. In their study, a lower bound to assess the independence of co-actors was computed for a collaborative visual search task (Brennan and Enns, 2015) using a race model (Miller, 1982). Brennan and Enns (2015) reasoned that having such a simulated lower bound of performance is a more appropriate lower bound than comparing performance to the individual performance of the better member of a pair (i.e., a lower bound used to assess collective benefits Bahrami et al., 2010). In particular, Brennan and Enns (2015) argued that a collective benefit can in principle be achieved with members of a pair acting independently simply due to the fact that two people perform a task. Therefore, we additionally estimated a pair performance based on the individual performances under the assumption that members of a pair act independently (termed “independent”). That is, the number of overlapping selections of individuals and whether these overlapping selections are correct or incorrect would randomly vary from trial to trial as participants would not intentionally select objects that systematically overlap or do not overlap. For the purpose of simulating the independent performance, for each trial of each pair, we took the hits and false alarms of each member and randomly distributed these among the targets and distractors. Based on these randomly distributed hits and false alarms, we computed a hypothetical pair performance. We repeated this procedure a 1000 times, resulting in a distribution of pair performances for a particular trial sampled under the assumption that members of a pair act independently. As an estimate of the independent performance for each trial and each pair, we took the mean of the simulated distribution of pair performances. By simulating such an additional lower bound of performance under the assumption that members of a pair act independently, the actual pairs' performances can be tested against this bound to assess whether members of a pair actually collaborated when they perform a task together (e.g., devise a collaborative strategy to distribute task demands).

As a point of note, the lower limit, upper limit, and independent performance are based entirely on the individual performances of members of a pair and not on the pairs' performances.

As a measure of how well co-actors divided task demands, we calculated the overlap for the target selections (i.e., how many object selections of members of a pair overlap) for each trial and divided this measure by the total number of selections.

### 2.5. Sliding window

To analyze our dependent variables across time, we performed a sliding window for each pair. In particular, as a first window, we took the data from the first ten trials of the experiment and calculated the mean across these trials and replaced the value of the first trial by that mean. We then shifted this window always by one trial (e.g., for the next step, we would use trials two to eleven) and repeated this procedure up to the 91st trial.

### 2.6. Cluster permutation tests

In order to assess whether performances differed significantly across time and between experiments, we used cluster permutation tests (Maris and Oostenveld, 2007). That is, given that we are interested in *when* pairs' performances reach a collective benefit, surpass the independent lower bound of performance, and differ between experiments, comparisons between conditions for each trial would be required. However, such a high number of comparisons would result in a high number of false positives, requiring the need to correct for multiple comparisons. Cluster permutation tests circumvent the need to correct for multiple comparisons as they take into account the relation between adjacent time points (i.e., trials in the present study) and statistical tests are performed on clusters (i.e., adjacent time points that exceed a critical value are grouped in one cluster) (Maris and Oostenveld, 2007). For a cluster permutation test, we first calculated the maximum number of temporally adjacent trials for which *t*-values with the same sign exceeded the critical *t*-value of significance. This maximum number constituted the largest cluster in the data. We also repeated this procedure to find the second largest cluster. As a point of note, for computing the *t*-values, if the comparison was a within-subject comparison (e.g., comparing the pairs' performances to the independent condition), we used the formula of a dependent *t*-test. For between-subject comparisons (e.g., comparisons across experiments), we used the formula for an independent *t*-test to compute the *t*-values. Finally, for comparisons of the pairs' data with a constant, we used a one sample *t*-test.

In order to assess the probability of cluster sizes occurring by chance, we simulated a hypothetical null distribution of cluster sizes under the assumption that there are no differences between the compared conditions. In particular, for within-subject comparisons, we randomly reassigned condition labels within each pair (e.g., whether the data belongs to the “independent” or “pair” condition) and calculated the largest cluster in this randomized data using the approach outlined above (i.e., grouping temporally adjacent trials in a cluster for which *t*-values with the same sign reach significance). For a between-subject design involving comparisons across experiments, we randomly assigned pairs to the experiments that are compared. For comparisons of the pairs' data with a constant, we randomly assigned condition labels (i.e., “constant" or “pair") within each pair. This procedure was repeated a 1,000 times with each iteration yielding the largest cluster in the randomized data. As a result, we created a null distribution of cluster sizes that was sampled under the assumption that there are no differences between the compared conditions.

To evaluate the significance of the largest cluster and the second largest cluster in the actual data, the *p*-values of these clusters were computed by calculating the fraction of clusters in the null distribution that were larger than the largest and second largest cluster in the actual data, respectively. If this fraction was below 0.05, a cluster in the actual data was deemed significant.

For all comparisons using a cluster permutation test, we report the extent of a cluster (range of trials), the *p*-value, and as an effect size Cohen's *d* averaged over the trials within a cluster (i.e., for each trial comparison, a separate Cohen's *d* is calculated). We chose Cohen's *d* as an effect size measure as it provides a normalized measure of the effect (i.e., standard deviation units) without taking the sample size into account. Note, depending on the type of comparison (i.e., whether it is a within-subject or between-subject comparison, or comparison with a constant), we used the appropriate numerator and denominator for the Cohen's d calculation. For a within-subject comparison, we used the standard deviation of the differences. For a between-subject comparison, we used the pooled standard deviation and for a comparison with a constant, we used the standard deviation of the group that is compared with the constant.

As a point of note, the extent of the clusters will not be interpreted in an absolute sense (Maris and Oostenveld, 2007). That is, we do not interpret the extent of a cluster within an experiment as the cluster sizes are dependent on several pre-selected factors (e.g., chosen critical value for the t-statistic, number of trials, number of participants). The extent of clusters within each experiment will only be interpreted in relation to the extent of clusters in the other experiments as the pre-selected factors influencing the cluster sizes are kept constant across experiments.

## 3. Results

In order to assess whether pairs reached a collective benefit and to what extent it differed across experiments, we first analyzed the pairs' and individuals' performances in each experiment and across experiments (see subsection “Collective Benefits” below). For assessing how effectively members of a pair divided task demands depending on the available information and their strategy-use, we then investigated the pairs' target selections and to what extent these overlap. Moreover, we assessed which type of division of labor strategies participants described in the questionnaire on strategy-use and whether the description fits to what participants did in the experiments (see subsection “Task Division & Strategy-use” below).

### 3.1. Collective benefits

**Figure 2:**
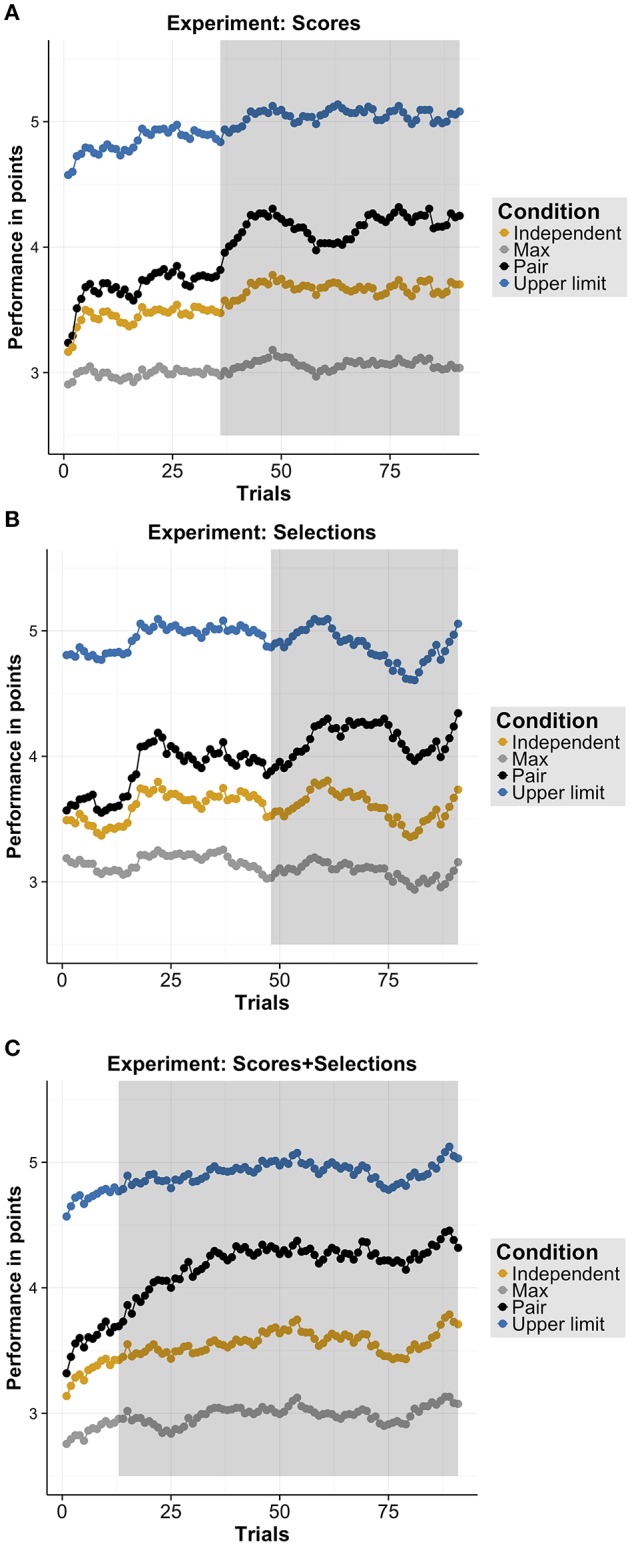
**Results overview. (A–C)** Performance in points for each measure (i.e., “Independent,” “Max,” “Pair,” “Upper limit”), separately for each Experiment: **(A)** Scores (Experiment 1), **(B)** Selections (Experiment 2), and **(C)** Selection+Scores (Experiment 3). Shaded regions indicate significant clusters using cluster permutation tests for comparing the pair's performance with the independent performance.

For each experiment, we analyzed whether the pairs' performances reached a collective benefit and also exceeded the estimated independent performance (i.e., a higher pair performance than max and independent; for a descriptive overview, see Figures 2A–C) using cluster permutation tests (for more details, see subsection “Cluster Permutation Tests” above). When pairs only received the performance scores (Experiment 1), pairs reached a collective benefit early (trials 3-91, *p* < 0.001, Cohen's *d* = 1.43) and over time exceeded the independent performance (trials 35–91, *p* < 0.001, Cohen's *d* = 0.86). We found similar results for the other two experiments. In particular, when pairs received only the partner's selections (Experiment 2), pairs also reached a collective benefit early (trials 2–91, *p* < 0.001, Cohen's *d* = 1.30) and exceeded the independent performance over time (trials 56–91, *p* = 0.003, Cohen's *d* = 0.88). In Experiment 3 (see Figure 2C), pairs received both the information of Experiments 1 and 2, they also reached a collective benefit early (trials 1–91, *p* < 0.001, Cohen's *d* = 1.23) and exceeded the independent performance (trials 16–91, *p* = 0.01, Cohen's *d* = 1.20). In sum, in each experiment, pairs reached a collective benefit and also exceeded the estimated independent performance. Comparing the extent of clusters across experiments, the collective benefit was reached early in each experiment while the pairs' performances exceeded the independent performance earlier in Experiment 3 than in Experiment 1 and 2 (see extent of significant clusters as gray areas in Figures 2A–C).

We also investigated in which experiment the pairs' performances stabilized the quickest (i.e., pairs did not improve their performance any further). For this purpose, we compared the pairs' performances in the last trial with the pairs' performances in the preceding trials using cluster permutation tests. For Experiment 1 (“Scores”), we found an early cluster (trials: 1–37, *p* = 0.001, Cohen's *d* = 0.87) and for Experiment 2 (“Selections") we also found an early cluster (trials 1-17, *p* = 0.009, Cohen's *d* = 0.76) as well as a later cluster (trials 43–52, *p* = 0.033, Cohen's *d* = 0.75). For Experiment 3 (“Selections+Scores"), we found that pairs' performances significantly differed from the last trial in an early cluster (trials: 1–19, *p* = 0.009, Cohen's *d* = 0.79). Overall, the extent of the cluster in each experiment suggests that pairs' performances stabilized the quickest in Experiment 3 followed by Experiment 1 and 2.

**Figure 3:**
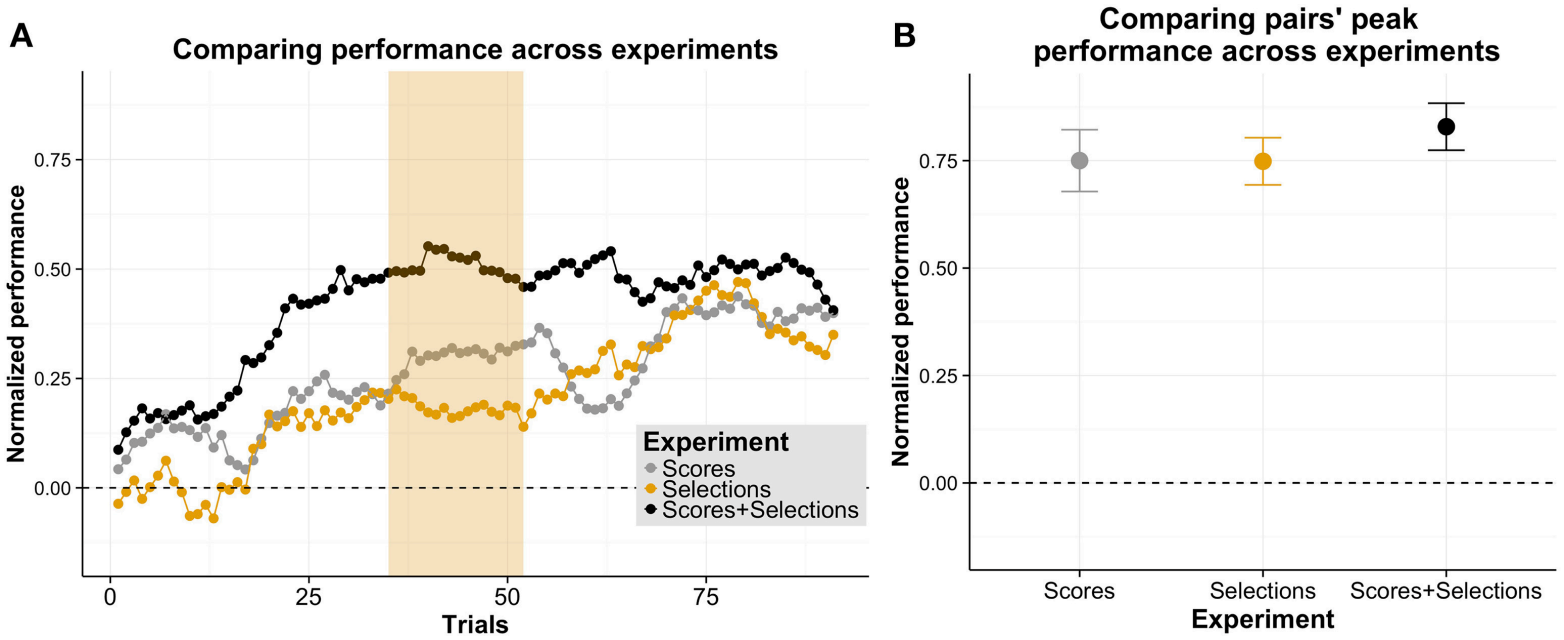
**Comparison of experiments. (A)** Normalized performance as a function of trials, separately for each of the three experiments (Experiment 1 “Scores”; Experiment 2 “Selections”; Experiment 3 “Scores + Selections”). Pairs' performances are normalized relative to the independent and the upper limit performances. The yellow shading indicates significant comparisons between Experiment 3 and 2. **(B)** Mean peak performance of each pair as a function of experiment. The error bars are standard error of the mean.

In order to investigate which type of information provided in the experiments led to the highest pair performances, we compared for which trials the pairs' performances across experiments differed. In order to account for systematic differences between experiments due to different levels of individual performances, we normalized the pairs' performances in each experiment relative to the independent condition and the upper limit of performance (for a descriptive overview, see Figure 3A). On a descriptive level, pairs that received both types of information (Experiment 3) reached a higher performance earlier than pairs in the other two experiments. However, over time, the pairs' performances converged to similar levels of performance. We tested whether these observations are statistically reliable using cluster permutation tests. When comparing Experiment 1 with 2 or 3, we found no significant cluster. When comparing Experiment 3 with 2, we found a significant difference with a larger extent (trials: 35–52, *p* = 0.030, Cohen's *d* = 0.24). In sum, these comparisons suggest that pairs reached a higher performance in Experiment 3 than in Experiment 2. However, this performance advantage in Experiment 3 was not sustained over the course of the experiment as no significant clusters were found for later trials. In order to investigate this observation in more detail, we additionally tested with a one factorial between-subject ANOVA whether the pairs' peak performances differed across experiments (see Figure 3B for a descriptive overview). We found no significant difference between performances [*F*_(2, 45)_ = 0.57, *p* = 0.570]. These data suggest that pair's performances' converged to similar levels later in an experimental session.

Overall, we found that pairs in all experiments reached a collective benefit and exceeded the estimated independent performance when performing the MOT task together. Moreover, we found that pairs' performances stabilized earlier when receiving both types of information (i.e., scores and the partner's selections, Experiment 3) than when only receiving the scores (Experiment 1) or the partner's selections (Experiment 2). Pairs also reached a higher performance in Experiment 3 than in Experiment 2 at first. However this performance advantage was not sustained over time. That is, performances converged to similar levels toward the end of the experiment.

### 3.2. Task division & strategy-use

**Figure 4:**
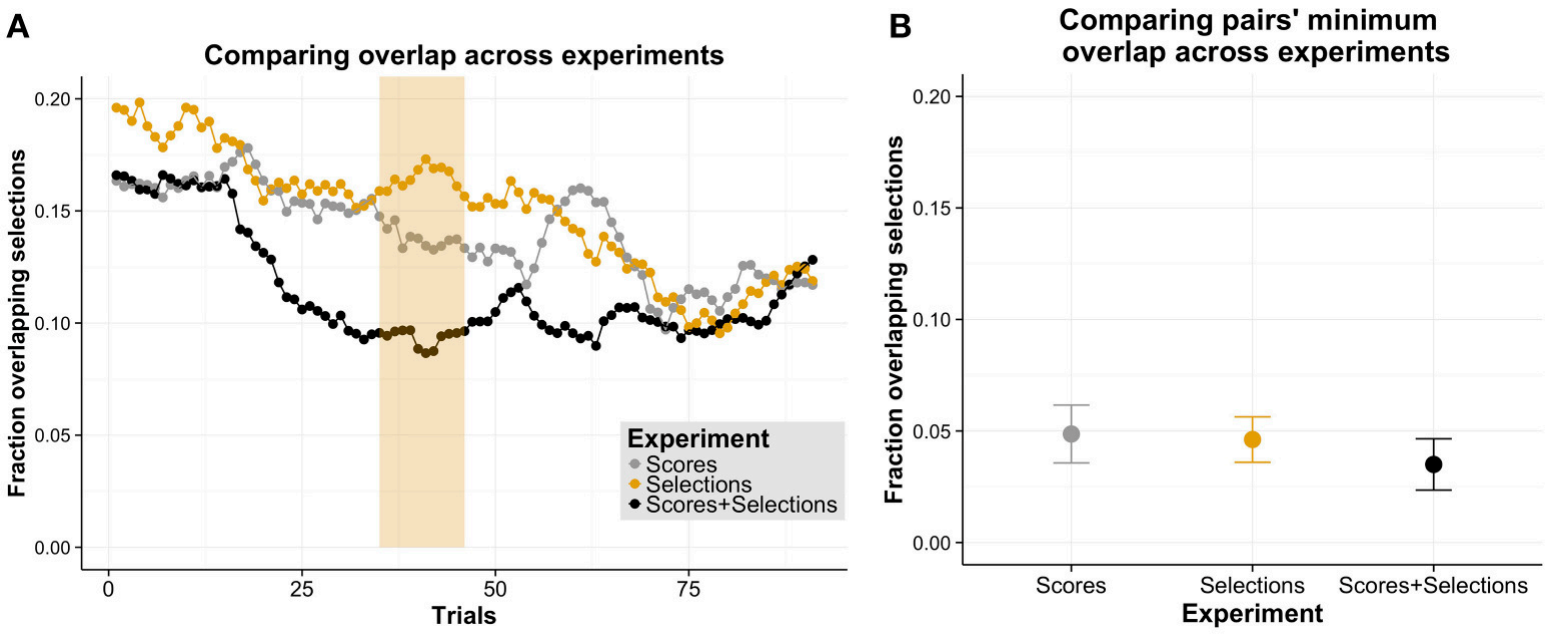
**Comparison of overlapping selections across experiments. (A)** Fraction of overlapping selections as a function of trials, separately for each experiment (Experiment 1 “Scores”; Experiment 2 “Selections”; Experiment 3 “Scores + Selections”). The number of overlapping selections are normalized relative to the total number of selections. The yellow shading in panel **(A)** indicates significant comparisons between Experiment 3 and 2. **(B)** Mean minimal fraction of overlapping selections as a function of experiments. The error bars are standard error of the mean.

In order to assess how effectively members of pairs divided the task demands, we investigated the fraction of overlapping selections (i.e., number of overlapping selections divided by the total number of selections) across experiments (see Figure 4A). Analogous to the comparisons above involving the pairs' performances across experiments, on a descriptive level, the fraction of overlapping selections are reduced early on in Experiment 3 and gradually in Experiment 1 and 2, converging to similar levels later in the experiments. We compared the fraction of overlapping selections across experiments using cluster permutation tests. We found no significant cluster when comparing Experiment 1 with Experiment 2 or 3. When comparing Experiment 3 with 2, we found a significant difference for a cluster with a larger extent (trials: 35–46, *p* = 0.034, Cohen's *d* = 0.23). These comparisons suggest that pairs reached a lower fraction of overlapping selections in Experiment 3 compared to Experiment 2. However, results also suggest that this difference is only present relatively early in the experiment as no significant clusters were found for later trials. In order to investigate this observation in more detail, we tested with a one factorial between-subject ANOVA whether the minimum fraction of overlapping selections of each pair differed across experiments (see Figure 4B for a descriptive overview). We found no significant difference for this measure [*F*_(2, 45)_ = 0.39, *p* = 0.678], suggesting that pair's fraction of overlapping selections converged to similar levels.

In sum, when comparing the fraction of overlapping selections across experiments, similar to our analysis of the performance above, we found that pairs in Experiment 3 had a lower fraction of overlapping selections than in Experiment 2. However, this difference was not found in later trials and also not when comparing the minimum overlap for each pair.

In order to assess which type of strategies pairs used to divide task demands, we analyzed the participants' responses in the questionnaire about their strategy-use. We found that participants described either one of two types of strategies which we termed a “left-right" division of labor strategy and “outer-inner” division of labor strategy, or no strategy at all. For the left-right strategy, participants described that they divided the targets into the left-most and right-most portion at the start of a trial. For the outer-inner strategy, participants described that one of the participants tracked the targets that were located more in the center of the display at the start of a trial while the partner would track the targets that were further away from the center.

Analyzing the fractions of these responses, for Experiment 1, we found that participants were predominantly described a left-right strategy (53.125%), followed by the outer-inner strategy (25%), with the fewest (21.875%) describing no strategy at all. For Experiment 2, we found that participants would only either describe the left-right strategy (75%) or no strategy at all (25%). For Experiment 3, we found that a left-right strategy was described by the most (75%) followed by the outer-inner strategy (15.625%) and no strategy (9.375%). We tested whether these observed differences were statistically reliable using a 3 × 3 χ^2^ test with the factors Strategy (left-right, outer-inner, none) and Experiment (Scores, Selections, Scores+Selections). We found a significant effect (χ^2^ = 11.38, *p* = 0.023), suggesting that the distribution of strategies differed across experiments, indicating that participants predominantly used a left-right strategy to collaborate with their partner. However, the use of a such a strategy was higher in the experiments in which the partner's selections were received (Experiments 2 and 3) than in an experiment in which only performance scores were received (Experiment 1).

In addition, we also compared the normalized performance between pairs that described a left-right strategy with pairs that either described an outer-inner strategy or no strategy in a 2 (Strategy) × 3 (Experiment) between-subject ANOVA. We found that pairs which described a left-right strategy performed significantly higher than pairs with an outer-inner strategy or no strategy [*M*_left-right_ = 0.36 vs. *M*_other_ = 0.10; *F*_(1, 42)_ = 6.44, *p* = 0.015]. We neither found a main effect of Experiment [*F*_(2, 42)_ = 0.38, *p* = 0.176] nor an interaction effect between the factors Strategy and Experiment [*F*_(2, 42)_ = 0.352, *p* = 0.705].

For Experiment 3, we additionally asked whether participants relied more on the partner's selections, scores, or both to develop their strategy. Participants indicated that they relied the most on the selections (50%) followed by scores (23.333%) and receiving both (26.666%). These results indicate half of the participants of Experiment 3 relied on the information about the actions of their co-actor to form strategies despite the fact that they have both types of information available.

**Figure 5:**
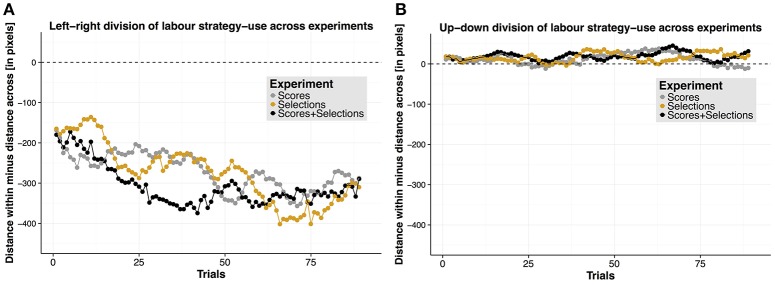
**Division of labor strategies. (A)** Left-right division of labor strategy. Horizontal distance difference as a function of trials, separately for each experiment. **(B)** Up-down division of labor strategy. Vertical distance difference is shown as a function of trials, separately for each experiment.

Given such a high prevalence for a left-right division of labor strategy in the questionnaire data, we investigated whether pairs actually performed such a strategy. Given members of a pair used a left-right division of labor strategy, we reasoned that the initial object positions of members' own target selections should be closer together than the distance of target selections across members. For calculating this difference, we first calculated for each trial and each member of a pair the horizontal distance (in pixels) between the initial positions of their individually selected targets and averaged across these values for each trial – this measure will be referred to as “distance within.” We then calculated the distance between the initial positions of the target selections *across* the selections of members of a pair (“distance across”). In order to have our final measure, we subtracted the distance across from the distance within values for each trial. As noted above, if members of a pair would use a left-right division of labor strategy, then we would expect a higher distance across value than distance within value, resulting in a negative residual. For this measure, on a descriptive level (see Figure 5A for an overview), we found a negative difference, suggesting that participants actually used a left-right division of labor strategy. We tested whether the calculated differences deviated significantly from zero using cluster permutation tests and found this to be the case for all experiments for clusters extending across all trials (Experiment 1: *p* < .001, Cohen's *d* = 1.33; Experiment 2: *p* < 0.001, Cohen's *d* = 1.49; Experiment 3: *p* < 0.001, Cohen's *d* = 1.38). These data converge on the conclusion that pairs actually applied a left-right division of labor strategy. We found no significant cluster permutation tests when we compared this measure across experiments (*ps* ≥ 0.24).

In order to validate whether the chosen measure is an appropriate one to characterize division of labor strategies, we repeated the procedure above for the vertical distance instead of the horizontal distances (see Figure 5B for an overview). Here, a negative residual would indicate that participants tended to divide the targets along the vertical dimension (i.e., chose an “up-down” division of labor strategy). As participants did not indicate in the questionnaire to have divided task demands along the vertical dimension, we expected no systematic differences between “distance within” and “distance across” for each experiment. That is, systematic differences would only occur if the “distance within” and “distance across” measures were different regardless of whether participants used an “up-down” division of labor strategy or not. We found no significant cluster permutation tests within each experiment (*ps* = 1) and across experiments (*ps* ≥ 0.12), suggesting that our measure to quantify left-right division of labor strategies, and the conclusion stemming from it, was valid.

## 4. Discussion

In the present study, we investigated how receiving information about actions of a co-actor, performance scores, or both contribute to the collective benefit in a collaborative visuospatial task. In contrast to earlier studies that did not experimentally manipulate the availability of these two types of information (Brennan et al., 2008; Brennan and Enns, 2015; Wahn et al., 2016c), we systematically varied whether members of a pair received performance scores, only information about the actions of their co-actor, or both. We found that these types of information either alone or in combination enable pairs to achieve a collective benefit early on. Furthermore, in each experiment, pairs also surpassed the performance predicted if members of a pair acted independently, suggesting that pairs did indeed collaborate to improve their performance (i.e., they effectively divided task demands).

In addition, participants' subjective reports on strategy-use further corroborate the conclusion that members of a pair collaborated in the task, as the majority of participants reported to have used a division of labor strategy (i.e., either a left-right or outer-inner division of labor). The most prevalent strategy that was reported across experiments was a left-right division of labor strategy (i.e., one co-actor would always track the leftmost targets while the other co-actor the rightmost targets), and we objectively confirmed that pairs actually used such a strategy. Earlier studies on collaborative visual search also found that pairs devised spatial division of labor strategies as well (Brennan et al., 2008; Brennan and Enns, 2015; Wahn et al., 2016c). Our present findings suggest that co-actors in collaborative visuospatial tasks generally prefer to use left-right division of labor strategies. As another point of note, in the present study subjective reports of a left-right devision strategy were particularly prevalent when participants were provided with information about the co-actor's target selections, suggesting that information about the actions of co-actors especially foster the formation of a left-right division of labor strategy.

When comparing the performance across experiments, we found that pairs reached a significantly higher performance early on when receiving both performance scores and information pertaining to the partner's selections than when only receiving either the performance scores or the partner's selections. However, this performance advantage was not found for later trials, suggesting that the pairs' performances converged to similar levels over time. A comparison of the peak performances across experiments also revealed no significant difference across experiments. These results were further supported by a significantly lower fraction of overlapping selections early on when receiving both performance scores and the partner's selections in comparison to only receiving the partner's selections. In sum, these findings suggest that pairs that received both types of information devised an effective collaborative strategy early on that was not further improved in subsequent trials. In particular, we suspect that the effectiveness of devised strategies could be verified quickly using the available information on performance scores and the number of overlapping selections, enabling pairs to divide the task demands quickly and effectively.

When only information about the co-actor's actions was available, pairs could only use the information about the overlapping selections as a means to verify their strategies, possibly slowing down the formation of effective division of labor strategies. In particular, the information about the partner's selections only informs participants about the number of overlapping selections but does not inform them whether their selections were actually correct. However, the fact that pairs in this experiment ultimately devised equally effective strategies relative to the devised strategies in the other two experiments indicates that pairs' selections over time do become more accurate. More generally, if information about the actions of the partner is available to co-actors in a collaborative spatial task, findings suggest that performance scores are not strictly necessary to devise effective devision of labor strategies.

Conversely, when only performance scores were available, participants could only use the available performance scores to verify whether the effectiveness of their division of labor strategy is increasing or decreasing but do not have information available on the actions of their partner. Members of a pair can only hypothesize how the division of labor strategy is implemented (i.e., which targets are tracked by the partner). However, again, the fact that pairs' devised strategies were equally effective relative to the strategies devised in the other two experiments suggests that receiving information about the actions of the partner is not strictly necessary to devise effective division of labor strategies. In short, the findings suggest that performance scores about the individuals' performances and the pair's performance are sufficient to devise effective division of labor strategies in collaborative spatial tasks.

In sum, having either of the two types of information (i.e., the partner's selections or the performance scores) is sufficient to devise an effective devision of labor strategy. Yet, having both types of information speeds up the development of such strategies.

Similar findings were found in earlier studies investigating collaborative decision-making tasks (Bahrami et al., 2012a). That is, pairs' performances were higher when they received performance scores in addition to exchanging information verbally compared to when they could only exchange information verbally without receiving any performance scores but converged to similar levels of performance over time (Bahrami et al., 2012a). Here, we found that in a collaborative visuospatial task, performance scores in addition to exchanging information about the co-actor's actions increased the pairs' performances at first and then converged to similar performance levels across experiments as well. More generally, these findings suggest that pairs in a collaborative task benefit from having an objective reference available to assess their performance.

Regarding division of labor strategies, a direction for future research is to identify the factors that may modulate the type of division of labor strategies that co-actors devise. For instance, the prevalence of left-right division of labor strategies could be biased by the shape of computer monitors that are used in studies investigating collaborative visuospatial tasks. In particular, monitors with a rectangle shape (i.e., with a larger width than height) were used in this study and earlier investigations on collaborative visuospatial tasks (Brennan et al., 2008; Brennan and Enns, 2015; Wahn et al., 2016c). For instance, it would be interesting to determine if left-right division of labor strategies vary in strength for quadratic stimulus displays and possibly flip to top-bottom strategies for rectangle displays with a greater height than width. More generally, traits as handedness (i.e., whether the participant is right or left handed) or the reading direction (i.e., whether the participant is a left-to-right or right-to-left reader) (Afsari et al., 2016) could also influence and/or facilitate the formation of strategies. That is, participants with opposite handedness' or reading directions may develop effective left-right strategies earlier.

Another possible direction for future studies could be to investigate whether division of labor strategies of similar effectiveness could be devised by decreasing the received information or replacing it by other information. Such studies would be of interest to investigate the minimal amount of information that needs to be exchanged between co-actors in a visuospatial task to devise effective division of labor strategies. In particular, in the present study the performance scores constituted feedback about the individual performances as well as the pair's overall performance. A future study could investigate the effectiveness of division of labor strategies when only a score about the pair's overall performance is available and no information about the individual performances is received. In particular, having no means to verify the accuracy of the individual selections might slow down the development of effective division of labor strategies and could modify the overall effectiveness of these strategies. Conversely, having only a score about the pair's performance available might be sufficient to devise an effective division of labor strategy, rendering individual performance scores unnecessary.

Another point to consider is that in the present study the information exchanged between co-actors of a pair was only given at the end of a trial. Future studies could investigate how the development of division of labor strategies is affected by exchanging information while simultaneously performing the collaborative MOT task. In particular, earlier studies on collaborative visual search tested to what extent the online exchange of spatial information about the actions of co-actors contributed to the collective benefit and found that this led to effective division of labor strategies (Brennan et al., 2008; Brennan and Enns, 2015; Wahn et al., 2016c). Similar to these studies, spatial information about the actions of co-actors (e.g., gaze information or verbal information) could be exchanged while participants track the objects in the MOT task. However, given findings of other studies investigating individual visuospatial processing capacities (Wahn and König, 2015a,b, 2016, 2017), processing spatial information about the actions of co-actors in addition to performing the MOT task could possibly interfere with performance, as both these types of information draw from a common pool of visuospatial attentional resources.

More generally, findings of the present study dovetail with other research that investigated the exchange of task-relevant information between co-actors in joint tasks (e.g., see: Knoblich and Jordan, 2003; Konvalinka et al., 2010, 2014; van der Wel et al., 2011; Fusaroli et al., 2012; Vesper et al., 2013, 2016b; Fusaroli and Tylén, 2016). That is, with regard to collective benefits, co-actors' joint performance is also facilitated by an exchange of information about the co-actors' task contributions in joint visuomotor tasks (Knoblich and Jordan, 2003; van der Wel et al., 2011) or in a joint perceptual decision-making task (Bahrami et al., 2010, 2012a; Fusaroli et al., 2012; Fusaroli and Tylén, 2016). Moreover, depending on the type of information that is exchanged between co-actors, co-actors systematically use different coordination mechanisms (Konvalinka et al., 2010, 2014; Vesper et al., 2016b). Relatedly, we find that the distribution of the used type of division of labor strategies changes depending on which type of information is exchanged between co-actors (i.e., information about the actions of co-actors, performance scores, or both).

From a more applied perspective, the present findings are applicable to circumstances in which humans need to perform demanding collaborative visuospatial tasks that are time-critical and/or only allow a very limited exchange of information between co-actors. Many professions place a high demand on visuospatial attention and at the same time require individuals to interact and cooperate. For instance, air-traffic controllers jointly need to track the trajectories of multiple airplanes on a screen. In such circumstances, it could be beneficial to only exchange the minimum amount of information necessary to devise an effective division of labor strategy, leaving more spare visuospatial attentional resources to perform the tracking task. Similarly, it would be beneficial for a security team tracking the position of several suspects in a large crowd to effectively divide task demands with only a minimum exchange of information, again leaving spare visuospatial attentional resources available to perform the tracking task more effectively. Moreover, the present findings are potentially applicable to scenarios, in which humans and robots jointly perform tasks (Schubö et al., 2007; Vesper, 2014; Ghadirzadeh et al., 2016). That is, the present study may provide indications which type of information is crucial for developing effective devision of labor strategies in collaborative visuospatial tasks that are jointly performed by humans and robots.

## 5. Author contributions

Study Design: BW, PK, and AK. Data Acquisition: BW. Data Analysis: BW. Wrote the manuscript: BW. Revised the manuscript: BW, PK, and AK.

## 6. Funding

We gratefully acknowledge the support by H2020 - H2020-FETPROACT-2014 641321 -socSMCs (for BW and PK), and the Quinn Exchange Fellowship (for BW). Moreover, we acknowledge support from the Deutsche Forschungsgemeinschaft (DFG), and Open Access Publishing Fund of Osnabrück University.

### Conflict of interest statement

The authors declare that the research was conducted in the absence of any commercial or financial relationships that could be construed as a potential conflict of interest.
